# The impact of digital economy on rural revitalization: Evidence from Guangdong, China

**DOI:** 10.1016/j.heliyon.2024.e28216

**Published:** 2024-03-28

**Authors:** Xueqin Deng, Mingshan Huang, Rong Peng

**Affiliations:** a Institute of New Development, Guangdong University of Finance and Economics, Guangzhou, China; b Guangdong Rural Development Research Center, Guangdong University of Finance and Economics, Guangzhou, China; c School of Economics, Guangdong University of Finance and Economics, China

**Keywords:** Rural revitalization, Digital economy, Nonlinear characteristics, Spatial effects

## Abstract

**Background:**

Digital technology and economy have profound impact on rural revitalization and become important initiatives to promote rural development. This study analyzes the spatial effect of digital economy on rural revitalization in Guangdong, China.

**Method:**

The entropy weight method is used to evaluate the level of rural revitalization and digital economy in 20 prefecture level cities of Guangdong province during 2011–2021. Spatial panel model is used to analyze the spatial spillover effect of digital economy on rural revitalization. Threshold model is employed to analyze the marginal effects of the digital economy on rural revitalization. Heterogeneous analysis is made by grouping the prefecture-level cities into four regions: the Pearl River Delta (PRD), the East Wing, the West Wing and the Mountainous region.

**Results:**

Both the level of rural revitalization and digital economy development in every prefecture-level city experienced an increase during 2011–2021 and the PRD region take the lead in both the rural revitalization and digital economy. There is significant spatial spillover effect of digital economy on the rural revitalization as a whole (the coefficient is about 0.3537). However, in the regions with a low level of digital economic development, there is a negative impact (−0.1847) of the digital economy on rural revitalization; while in regions with medium or high level of digital economy, it has a significant positive effect (0.3835) on rural revitalization. The impact of digital economy on rural revitalization development is significantly greater in the PRD (0.3141), the East Wing (0.7215), the West Wing (0.5467) than in the mountainous region (0.2238).

**Conclusions:**

There is significant spatial differentiation in the digital economy and rural revitalization. The digital economy has significant spatial spillover effect on the rural revitalization. And this effect is found out to have heterogeneous and non-linear characteristics. In order to promote rural revitalization, it is necessary to make full use of the digital economy and technology in rural areas. Besides, it is important to improve the digital skills and literary of the rural population so that the digital economy could play a greater role in rural revitalization.

## 1 Introduction

After decades of reform and opening up, China's economy has realized tremendous development, with per capita GDP growing from 100,280.1 billion yuan in 2000 to 1,143,669.7 billion yuan in 2021, at an average annual growth rate of 8.6% [1]. At this stage, one of the most prominent contradictions is the huge difference and imbalance between urban and rural development. In 2021, the per capita disposable income of urban residents was 2.5 times that of the per capita disposable income of rural residents, which was 47,412 yuan for urban residents and 18,931 yuan for rural residents [1]. The pace of rural development must be accelerated to solve this problem. The 2017 report of the 19th National Congress put forward the strategy of rural revitalization, pointing out that priority should be given to the development of agriculture and rural areas, establishing and improving the institutional mechanism and policy system for the integrated development of urban and rural areas, and accelerating the modernization of agriculture and rural areas, in accordance with the general requirements of industrial prosperity, ecological livability, rural civilization, effective governance and an affluent life [2].

To accelerate the modernization of agriculture and rural areas, it is important to use digital tools and technologies in agriculture and rural areas and it is worth emphasizing that the digital economy is one of the key factors affecting rural and agricultural development. Digital technologies are powerful innovations that enhance operational improvements as well as social and environmental sustainability, and the digital economy is a key driver of agriculture and rural economy [[3], [4], [5], [6]]. First, the digital tools and digital technologies help to improve the productivity in agriculture sector and optimize traditional rural infrastructure [7]. Digital economies could help to promote the marketing of agricultural products and boost rural industrial development [3]. Second, the digital economy can be a way to optimize the rural ecosystem and environmental quality, for example, it can lead to significant reductions in fertilizer application [8] and pesticide [9]. Third, advanced information technologies could help to promote the inheritance of traditional rural culture and to develop creative industries in rural regions [10,11], making the countryside more cultured and civilized. Fourth, digital technology empowers rural self-governing organizations and the construction of digital villages makes rural governance more effective at four levels: infrastructure system, village brain, application support system and application service system [12]. Lastly, ICTs have facilitated off-farm employment for farmers [13] and the transformation of the rural economy, thereby increasing farmers' incomes [14] and eliminate poverty [15]. The growth of the digital economy has raised the standard of living of farmers and made rural residents more affluent [13,16]. Combining the above impacts of the digital economy on agriculture and rural areas, we are trying to measure the impact of the digital economy on rural revitalization through a spatial econometric model.

We take Guangdong Province as the case study to analyze the impact of digital economy on rural revitalization since it is not only one of the fastest growing provinces in China, but also a region where there is a great imbalance between urban and rural development and regional development. In 2021, the per capita disposable income in Guangdong Province was 44,993-yuan, 54,854 yuan for urban residents and 22,306 yuan for rural residents respectively, with an urban/rural income ratio of 2.46. Looking at the different cities in the province, Guangzhou's per capita disposable income was as high as 68,908 yuan, while Jieyang's was only 23,781 yuan, with the former more than three times as high as the latter [17]. The imbalance in Guangdong's economic development is also reflected in the uneven development of villages in different geographical areas. In particular, most of the villages in the Pearl River Delta region have been industrialized and urbanized, and their income is not much different from that of cities, whereas the development of the rural areas located in the eastern, western and northern parts of Guangdong lags far behind that of the cities, resulting in a large urban-rural income gap. In order to narrow the urban-rural gap, the Guangdong Province decides to implement the “High quality Development Project for Hundred Counties, Thousand Towns, and Ten Thousand Villages” [18].

Guangdong Province is the largest province in terms of digital economy, with the scale of 5.9 trillion yuan in 2021, ranking first in the country for five consecutive years. The proportion of the digital economy to GDP has reached 47.50% [19]. The rapid economic growth, uneven development between urban and rural areas, and the rapid development of the digital economy have made Guangdong a typical representative of China's economic development. Therefore, this study attempts to study the effect of digital economy on rural revitalization by using panel data of Guangdong Province from 2011 to 2021, so as to provide Guangdong experience for rural revitalization in other regions, and provide new enlightenment for the designation of rural revitalization policies in China. The marginal contributions of this study are: Firstly, by reviewing existing literature on rural revitalization and digital economy, this study comprehensively measures the development level of rural revitalization and digital economy in Guangdong Province, and conducts a study on the relationship between the two and their temporal and spatial pattern evolution; Secondly, this study delves into the empowering effect of digital economy on rural revitalization in Guangdong Province and its nonlinear characteristics by constructing threshold models; Thirdly, based on the fixed effects spatial Durbin model, this study researches into the spatial empowerment and spillover effects of the digital economy on rural revitalization in Guangdong Province and explores the empirical research on digital economy's effect on rural revitalization.

## 2 Theoretical framework and research hypothesis

### 2.1 Theoretical framework

The essence of digital technology is to store and transmit information in bits. From an economics perspective, digital technology has brought various cost savings in production and other applications. Overall, cost savings can be reflected in five aspects:lower search costs, lower replication costs, lower transportation costs, lower tracking costs and lower verification costs [20].

The general requirements of the strategy of rural revitalization include five aspects: industrial prosperity, ecological livability, rural civilization, effective governance and an affluent life [2]. The cost-effectiveness of applying digital technology in rural areas could lead to the prosperity of rural industries, the ecological environment, the civilization of the countryside, effective governance and the improvement of the living standards of rural residents.

Reduced search costs, tracking costs and validation costs help promote rural industries and the realization of a prosperous life for rural residents. Reduced search costs facilitate quick matching of sellers and buyers thereby facilitating the marketing of agricultural products. Thanks to digital platforms, digital technology can reduce the cost for sellers to find buyers and it provides a wide market for the sale of agricultural products. Digital platform has enabled firms, even those rural suppliers located away from the major population and commerce centers, to prosper [21]. The digital technologies bring more opportunities to the rural business [22]. The use of e-commerce can make it possible to expand online business in remote areas and even sell agricultural products abroad at a lower cost, lifting rural residents out of poverty [15,21]. Digital technology has flattened the world, making it possible for people anywhere to connect to the world, and has facilitated the sale of agricultural products to overseas markets, and farmers' incomes have increased as a result. Thus, ICT adoption and Internet use have a significant positive impact on income diversification, especially the off-farm economic activities [23], thereby positively affecting farmers' incomes [24,25].

Lower tracking costs make it relatively easy to deliver advertisements to potential customers and it has also led to a reduction in costs associated with the verification of identity and reputation. Lower verification costs are very important for the marketing of agricultural products since it makes it easier to certify the reputation and trustworthiness of any seller in the digital economy. As small players in digital markets, various types of produce are not well known to customers. Reputation mechanism establishes trust between suppliers and customers, which is the basis for stable supply and demand relationship of agricultural products. Stabilized supply and demand are an important guarantee for the prosperity of agriculture and the key to raising the incomes of those working in agriculture. In rating systems of digital platforms, the ratings of buyers and sellers are published for reference for future market participants, which would serve as a reputation mechanism. Therefore, the lower tracking costs and verification costs help promote the sale of agricultural products through reputation mechanism of digital platform.

Besides, lower research cost enables more efficient financial markets and labor markets. The positions in rural areas could be matched with suitable labor resources, while attractive investment projects in rural areas can be matched with suitable investors. The digital technology in the financial market as well as in the job market facilitates the matching of factors of production in rural areas. The application of digital technology in the financial market as well as in the job market facilitates the matching of factors of production in rural areas. For example, the application of digital financial inclusion in China can enable more rural residents to obtain loans, which is conducive to the expansion of agricultural production. Job information is posted on the Internet, saving time for both job seekers and employers in the job market. The use of digital technology in the job market allows for quick matching of supply and demand between job seekers and employers, providing rural residents with more employment opportunities and sources of income. On the other hand, it has helped to find suitable talents for key positions for industrial development in rural areas.

The impact of digital technologies on agricultural development is also reflected in its application to improving agricultural production tools and techniques. Fruit picking robots, drones spraying insecticides (Costa et al., 2023), radio frequency identification (RFID) technology [4], artificial intelligence [6] and so on, are typical digital technologies to improve productivity in agricultural sector [3]. Digital capital has already had a greater impact on industry development than traditional capital [26].

Rural governance benefits from digital technology because reduced replication costs allow the information to be shared without increasing any costs. Effective communication between the government and rural residents has always been a challenge in traditional rural governance. Digital technology offers a good solution for this, namely, to provide information to the population through the Internet at no additional cost. The prominent feature of digital products is that they are non-rival, which means that they can be consumed by one person without reducing the amount or quality available to others [20]. Thus, digital technology provides a cost-effective way of communication between the government and rural residents. In addition, information between the government and rural residents could be delivered more effectively through the Internet and mobile terminals. The e-government system for handling administrative matters online saves rural residents a great deal of time.

The barriers to collaboration between government departments can be eliminated because the data resources of each department can be unified and integrated, and then they are shared and exchanged between government departments [27]. Digital technology also provides a channel for multi-party consultation and common governance, through which information can be updated and transmitted, preventing the monopolization of information. Rural residents are guaranteed the right to speak and act in rural governance, and their sense of democracy and rights is stimulated. Digital technology protects the right of rural residents to participate in governance, resulting in a “decentralized” model of rural governance. In practice, digital technology empowers rural self-governing organizations and the construction of digital villages makes rural governance more effective at four levels: infrastructure system, village brain, application support system and application service system [12]. [[22], [21], [20]].

Digital technology has brought about a reduction in transportation costs and tracking costs, allowing for the improvement of rural ecosystems. The almost 0 replication cost makes the storage cost of transportation information in bits over the Internet close to 0 as well. Digital technologies have been widely used in transportation, such as the Global Positioning System (GPS), which has significantly reduced transportation costs. The natural and ecological environment of the countryside is attractive for people living in cities, and as transportation becomes more convenient and the cost of transportation decreases, more and more people travel to the countryside. As a result, rural residents receive off-farm income. This has led to a greater emphasis on preserving rural ecosystems. On the other hand, digital technology has made it possible to reduce the use of fertilizers and pesticides, as its use in agricultural technology allows for the precise application of fertilizers [8] and pesticides [9]. Empirical studies have shown that there is an association between Internet use and environmentally friendly agricultural innovation adoption, which makes the agricultural development more sustainable [16]. The upgrading and automation of wastewater treatment plants through IoT [28] and the role of socio-cyber-physical systems in agriculture and the rural social environment [29] show the important impact of the digital economy on the rural environment [23,24].

The application of digital technology helps the development of rural culture and promotes rural civilization. The main reason why rural culture lags behind that of towns and cities is that rural areas do not have equitable access to knowledge, educational resources and other sources of information. But the reduced replication costs have broadened rural residents' access to knowledge and educational resources. The non-rivalrous nature of digital technology could enable rural residents to access the same information as people in cities, conditional on having access to the Internet. Rural consumers would benefit by having access to the same set of digital products and services as everyone else [20], For example, digital museums and digital libraries are cultural products that rural residents can access for free [27]. More online video courses also provide opportunities for rural children to enjoy more equitable education. On the other hand, traditional culture in rural areas can be better disseminated and passed on because of the reduced replication costs. For example, digitization is a better way to preserve intangible cultural heritage, i.e., preserving and managing intangible cultural heritage through digital technology, such as constructing a database on intangible cultural heritage [10,11]. [[25], [26], [27], [30], [31], [28], [29]].

Based on the preceding analysis, the following research hypothesis is proposed.

Hypothesis 1 There is a positive impact of the digital economy on the rural revitalization.

### 2.2 The digital economy has a non-linear impact on rural revitalization

Digital economic development tends to be spatially nonlinear [32]. Wang and Cen (2022) found that the nonlinear characterization of the increasing marginal effect of the digital economy on innovation efficiency [33]. Digitization and digital economic development have significant impacts on employment, household income, and investment, but these impacts vary across groups and regions due to differences in the availability of digital infrastructure, digital economic activities, levels of economic development, educational attainment and industry and employment structures [31,[34], [35], [36]].

The logic of the non-linear impact of the digital economy holds true for its impact on rural revitalization. ICT adoption plays a role in promoting rural income diversification and has a greater impact on low-income rural households [31]Due to the existence of demographic differences in various regions, there are demographic differences in the enabling effect of the digital economy on rural revitalization. In rural areas where the demographic structure is aging seriously, the differences in learning ability and education level lead to the digital divide, thus preventing the digital economy from playing a positive role in rural revitalization, and despite the injection of sufficient funds by government policies, it is still unable to fully unleash the driving force of the digital economy in rural revitalization; whereas, in rural areas where the degree of aging is less serious, the advantage of the learning ability of the younger generation has been brought into full play. They can quickly master the application methods of various digital products, enabling the digital economy to develop rapidly in rural areas and promoting the digital development of rural industries. The e-commerce platform to the countryside further amplifies the driving effect of the digital economy on rural revitalization. Thus, improving rural education and infrastructure, such as roads and broadband facilities, can help increase the adoption of ICTs by rural households [31], thus promoting rural revitalization.

Based on the analysis, the second research hypothesis is proposed.

Hypothesis 2 The effect of digital economy development on rural revitalization and development has non-linear characteristics.

### 2.3 The digital economy has spatial spillover effects on rural revitalization

The digital economy has significant spatial spillover effects [33,37]. The digital economy is characterized by being virtually independent of geographic and administrative boundaries, enabling the cross-regional allocation of factors of production [38], including manpower, capital, information and innovation [33]. For example, Internet platforms and inclusive finance to the countryside have enabled one region to have an impact on another, creating spatial spillover effects [39].

Because of the digital economy's nature of diminishing marginal cost, the digital economy exhibits the distinctive characteristics of economies of scale, economies of scope and long-tail effects [40]. Digital platforms generate economies of scale through positive network externalities. The value of a network depends on the number of users it connects, so the value of the network increases exponentially with the number of users [41]. Based on huge user resources, digital platforms can provide both various types and small quantities of products and services to meet the needs of “niches” and large quantities and single varieties of products and services to meet the needs of the general public, thus realizing economies of scope [40]. In a word, digital economy is built on the network externalities arising from the vast customer resources. It can be seen that when the development of the digital economy in one region brings about rural revitalization, neighboring regions will also drive their rural revitalization due to the network externality characteristics of the digital economy.

The digital economy helps to promote the development of industrial clusters in the countryside [42] because digital technology improves the efficiency of information transmission and the availability of factors such as capital and labor in the countryside industry. High concentration of similar or related industries in a specific region will result in agglomeration economy [43]. On the one hand, the theory of agglomeration suggests that industrial clusters have positive externalities [44]. Agglomeration in the same industry facilitates the sharing of knowledge, information, infrastructure, labor markets and intermediate product markets among firms and the strengthening of inter-firm linkages [44]. Industrial clusters have economic radiation effects. It drives the regional economic development of the surrounding areas through industrial transfer, technology diffusion and other ways. The neighboring regions could realize rural revitalization through the absorption of industrial clusters of external spillover effects.

Based on the analysis, the third research hypothesis is proposed.

Hypothesis 3 The digital economy has spatial spillover effects on rural revitalization.

## 3 Data and methods

### 3.1 Data sources

The Digital Inclusive Finance Index is derived from the Peking University Digital Inclusive Finance Index (2011–2021) compiled by the Digital Inclusive Finance Center of Peking University. The number of digital patents is sourced from the Chinese Research Data Services (CNRDS) database, while the remaining data is sourced from the “Guangdong Statistical Yearbook”, “Guangdong Rural Statistical Yearbook”, and the statistical yearbooks of various cities in Guangdong. With reference to Ref. [45], Shenzhen city was excluded because the proportion of Shenzhen's urban population in the permanent population reaches more than 99% from 2011 to 2021 according to the Guangdong Statistical Yearbook, indicating that Shenzhen has completed urbanization. Stata 16 is used for data processing and spatial model analysis. ArcGIS10.8 is used for mapping.

### 3.2 Variables

#### 3.2.1 Explained variable

Rural revitalization is the explained variable. In this study, we use an index system to measure rural revitalization which encompasses the five dimensions of “industrial prosperity, ecological livability, civilized rural customs, effective governance, and affluent living".

#### 3.2.2 Core explanatory variable

Digital economy is the core explanatory variable. We use an index system including four dimensions of “digital infrastructure [35], industrial digitization [46], digital industrialization and digital innovation” to measure the digital economy for each region.

#### 3.2.3 Control variables

The control variables in this study are: the level of urbanization, the government financial input and the regional government expenditure on agriculture, forestry and water conservancy facilities.

Level of urbanization is measured by the proportion of urban population to the total population. Since increased levels of urbanization are often accompanied by upgrades in rural industries and improvements in people's well-being, it is likely to lead to biased research results if this variable is not considered. Therefore, the urbanization level variable is used to control its impact on rural revitalization and development in this study.

Government financial input refers to the investment in the primary sector, measured by the share of investment in fixed assets in the primary sector in total investment in fixed assets. As one of the main sources of physical capital in the primary sector, the level of capital investment by the government has a significant impact on industrial development in rural areas. Therefore, it is necessary to control for this variable.

Expenditures on agriculture, forestry and water conservancy facilities are used to measure the share of regional expenditures in Guangdong Province's expenditures in these areas.

#### 3.2.4 3.3 Index systems3.3. 1 Index system for measuring the rural revitalization

The indicator system for measuring rural revitalization in the literature mainly focuses on the following five aspects: industrial prosperity, ecological livability, rural civilization, effective governance, and affluent living [47]. This is based on the goals that the central government should achieve for rural revitalization. Therefore, this study also evaluates rural revitalization through the above five aspects. Industrial prosperity is reflected in land productivity, agricultural mechanization level, industrial structure and coordination degree of industrial development. Ecological livability is reflected in fertilizer usage intensity, pesticide usage intensity, centralized sewage treatment rate, harmless treatment rate of household waste and per capita artificial afforestation area. Rural civilization is reflected in proportion of cultural and entertainment expenses, student/teacher ratio at secondary school and primary school. Effective governance is reflected in proportion of science popularization demonstration villages, coverage rate of agricultural machinery institutions, per capita collective assets, and proportion of social security and employment in fiscal expenditure. Affluent living is reflected in per capita disposable income of farmers, income ratio of urban and rural residents, Engel's coefficient, and proportion of villages with operating income. Please refer to Table 1 for the specific index interpretation for each indicator.

**Table 1:** Index system for rural revitalization.

Primary indexes	Secondary indexes	Index interpretation	Unit	Attributes
Industrial prosperity	Land productivity	Agricultural output value/Total cropped area	10000 yuan/hectare	+
	Labor productivity	Total output of agriculture, forestry, animal husbandry and fishery/number of employees in the primary industry	10000 yuan/person	+
	Agricultural mechanization level	Total agricultural machinery power/Main crop sowing area	Kilowatt/hectare	+
	Industry structure	Number of employees in the primary industry/number of employees in rural areas	%	–
	Coordination degree of industrial development	Primary industry output/GDP	%	–
Ecological livability	Intensity of fertilizer use	Fertilizer usage/sowing area of main crops	Tons/hectare	–
	Intensity of pesticide use	Pesticide usage/sowing area of main crops	Tons/hectare	–
	Concentrated sewage treatment rate	Centralized sewage treatment volume/total sewage discharge	%	+
	Harmless treatment rate of household waste	Harmless treatment amount of household waste/total amount of household waste	%	+
	Per capita artificial afforestation area	Artificial afforestation area/number of employees in the primary industry	Mu/person	+
Rural civilization	Proportion of cultural and entertainment expenditure	Cultural and entertainment expenditure/Per capita consumption expenditure	%	+
	Middle school student-teacher ratio	Number of middle school students/Number of full-time middle school teachers	%	–
	Primary school student-teacher ratio	Number of primary school students/Number of full-time primary school teachers	%	–
Effective governance	Proportion of popular science demonstration villages	Number of science popularization demonstration villages/total number of villages	%	+
	Coverage rate of agricultural machinery household institutions	Number of agricultural machinery institutions/number of employees in the primary industry	institution/1000 employees	+
	Per capita collective assets	Total assets of village collective/population of townships	Yuan/person	+
	Proportion of fiscal expenditure on social security and employment	Social security and employment expenditure/public budget expenditure	%	–
Affluent living	Per capita disposable income of farmers	Per capita disposable income of rural residents	Yuan/person	+
	Income ratio of urban and rural residents	Per capita disposable income of urban residents/per capita disposable income of rural residents	%	–
	Engel's coefficient	Food expenditure/per capita consumption expenditure	%	–
	Proportion of villages with operating income	Number of villages with operating income/total number of villages	%	+

#### 3.2.5 3.3.2 Index system for measuring the digital economy

The index system for measuring the digital economy usually includes digital infrastructure [33], industrial digitization [48], digital industrialization [48]and digital innovation [46]. With reference to the literature, the index system for measuring the digital economy is concerned with the following four aspects: digital infrastructure, industrial digitization, digital industrialization and digital innovation. Digital infrastructure is reflected in Internet penetration and cell phone penetration rate. Industrial digitization is reflected in Internet finance. Digital industrialization is reflected in telecommunications industry and practitioners in the digital industry. Digital innovation is reflected in digital innovation inputs and outputs. Please refer to Table 2 for the specific index interpretation for each indicator.

**Table 2:** Index system for digital economy.

Primary indexes	Secondary indexes	Index interpretation	Unit	Attributes
Digital Infrastructure	Internet penetration rate	Number of internet users/Resident population	%	+
	Mobile phone penetration rate	Number of mobile phone users/Resident population	%	+
Industrial Digitalization	Internet finance	Digital Inclusive Finance Index	–	+
Digital Industrialization	Post and telecommunications	Logarithm of per capita post and telecommunications services	–	+
	Employees in digital industry	Number of employees in Information Transmission, Software, and Information Technology Services	10,000 persons	+
Digital Innovation	Digital innovation investment	Per capita expenditure on science	Yuan/person	+
	Digital innovation output	Number of digital patents	Patents	+

### 3.3 Entropy weight method for calculating the index of rural revitalization and digital economy

The entropy method [49] is employed to calculate the level of rural revitalization and digital economy for each region. The following are the specific steps to calculate the index of rural revitalization and digital economy for each year.

Standardization of the index. Assuming there are m regions, n indicators, and T years, the data would be standardized in the following ways. The first is for positive index and the second is for negative one.

$${x_{\text{ij}}}^{\prime }=\frac{{x_{\text{ij}}}^{t}-\min\;\left\{{x_{\text{ij}}}^{t}\right\}}{\max\;\left\{{x_{\text{ij}}}^{t}\right\}-\min\;\left\{{x_{\text{ij}}}^{t}\right\}}$$

$${x_{\text{ij}}}^{\prime }=\frac{\max\left\{{x_{\text{ij}}}^{t}\right\}-{x_{\text{ij}}}^{t}}{\max\;\left\{{x_{\text{ij}}}^{t}\right\}-\min\;\left\{{x_{\text{ij}}}^{t}\right\}}$$

Where, ${x_{ij}}^{t}$ is the value of the indicator j in the region i of the year t; ${x_{ij}}^{\prime }$ is the *j*th indicator for the *i*th region in year *t*; $\max\;\left\{{x_{\text{ij}}}^{t}\right\}$ is the maximum value of the *jth* indicator of all the years; $m\text{in}\left\{{x_{ij}}^{t}\right\}$ is the minimum value of the *j*th indicator of all the years.

Calculate the proportion of index.

$${w_{ij}}^{t}=\frac{{x_{ij}}^{\prime }}{\sum_{t=1}^{T}\sum_{i=1}^{m}{x_{ij}}^{\prime }}$$

Where, ${w_{ij}}^{t}$ is the proportion of the *j*th indicator for the *i*th region in year t of all the years.

Calculate the information entropy of index.

$$E_{j}=-k\sum_{t=1}^{T}\sum_{i=1}^{m}{w_{\text{ij}}}^{t}\times \ln\;\left({w_{\text{ij}}}^{t}\right)$$

Where, $E_{j}$ is the information entropy value of the *j*th indicator. k=ln(mT).

Calculate the non-mixed information values of indicators

$$d_{j}=1-E_{j}$$

Where, $d_{j}$ is the non-mixed information value of the *j*th indicator.

Calculate the weight of index.

$$r_{j}=\frac{d_{j}}{\sum_{j=1}^{m}d_{j}}$$

Where, $r_{j}$ is the weight of the *j*th indicator in the comprehensive index

The composite index is calculated as follows:

$${{Index}_{i}}^{t}=\sum_{j=1}^{m}r_{j}\times {x_{ij}}^{\prime }$$

### 3.4 Models

#### 3.4.1 Baseline model

Equation (1) is the baseline model, which is used to verify that the digital economy has a significant positive effect on rural revitalization.

(1) $${Rural}_{it}=\alpha_{0}+\alpha_{1}{\text{Digit}}_{it}+\alpha_{2}{\text{Urban}}_{it}+\alpha_{3}{level\_ATW}_{it}+\alpha_{4}{\text{Capi}}_{it}+\mu_{it}$$

Where, ${Rural}_{it}$ is the level of rural revitalization for region i in period t, ${Digit}_{it}$ is the core explanatory variable in this study, which refers to the level of digital economy development of region *i* in period *t*. ${Urban}_{it}$ denotes the level of urbanization, ${level\_ATW}_{it}$ denotes the government financial inputs; ${Capi}_{it}$ is the amount of investment in fixed assets in agriculture, forestry, animal husbandry and fisheries, α is the coefficient, $\mu_{it}$ denotes an error term.

#### 3.4.2 Threshold model

Equation (2) is the threshold model which is employed to determine whether digital technology has a non-linear impact on rural revitalization, we need to verify the stability of coefficient estimates, that is, whether there is a turning point between the digital economy and rural revitalization, and whether it appears in the form of a piecewise function. Quantile regression and threshold models are commonly used methods for analyzing nonlinear effects. The quantile regression has a significant drawback, as it can only be estimated within the range of quantiles and the exact quantile value can't be determined. The threshold model proposed by Hansen in 1999 effectively solved this problem by determining the value of the threshold and providing a test of the existence of the threshold [50]. The following threshold model is constructed to analyze nonlinear effects [51].

(2) $${Rural}_{it}=\beta_{0}+\beta_{1}Z_{i,t}+\beta_{c}{\text{Digit}}_{it}\times I\left(\cdot \right)+\mu_{it}$$

Where, the vector $Z_{i,t}$ represents a series of control variables, I(·) is the threshold indicator function, which takes the value of 1 if it meets the condition in parentheses, and 0 otherwise, and the specific function form is determined after the threshold test; β is the coefficient to be estimated. The above single threshold model could be extended to a double threshold or three threshold forms based on the threshold test results.

#### 3.4.3 Spatial weight matrix

Spatial weight matrix (SWM) is a prerequisite for doing spatial regression analysis, which represents spatial data based on the relationship between each spatial location and is used to measure spatial correlation as well as to discover the spatial strength of spatial relationships [52].

Specifically, the proximity of spatial regions of n locations is expressed by defining a binary symmetric spatial weight matrix W. Three spatial matrices, namely, geographic adjacency matrix, geographic distance matrix, and geo-economic nesting matrix, are employed in the study.

Geographic adjacency matrix is expressed as follows:

$$W_{\text{ij}}=\left\{ \begin{array}{c} 1，\text{When}\;\text{region}\;i\;\text{is}\;\text{adjacent}\;\text{to}\;\text{region}\;j\\ \;0，\;\text{otherwise}\; \end{array}\right.\;\left(i,j=1,2,\ldots ,n\right)$$

Where, n denotes the number of regions, $W_{ij}$ denotes the element in row i and column j of the spatial weight matrix. $W_{ij}=0$ if i=j. A value of 1 or 0 is assigned when there is a neighboring boundary between the two regions, otherwise 0.

Geographic distance matrix calculates spherical distances based on latitude and longitude instead of straight-line distances in the traditional Cartesian coordinate system. It is calculated as follows:

$$W_{\text{ij}}=\left\{ \begin{array}{c} R*\text{arcos}\left[\sin\;\varphi_{i}*\sin\;\varphi_{j}+\cos\;\varphi_{i}*\cos\;\varphi_{j}*\cos\left(\tau_{i}-\tau_{j}\right)\right]，i\neq j\\ 0，i=j\; \end{array}\right.$$

i,j=1,2,…,n

Where, $W_{ij}$ denotes the spherical distance between region i and region j. $\varphi_{i}$ and $\tau_{i}$ denotes the latitude and longitude of region i.R denotes radius of the earth.

Geo economic nested matrix embeds economic factors in the geographic distance matrix. Specifically, the level of economic development of each region is included in the matrix so as to reflect the economic linkages between regions. It is calculated as follows:

$$W_{\text{ij}}^{\text{de}}=\left\{ \begin{array}{c} \frac{1}{|\omega_{i}-\omega_{j}+1|}\times e^{-W_{\text{ij}}}\;i\neq j\\ 0，\;i=j\; \end{array}\right.\;i,j=1,2,\ldots ,n$$

Where, $W_{ij}^{de}$ denotes the economic distance between region i and region j, $W_{ij}$ denotes the spherical distance between region i and region j. $\omega_{i}$ indicates the average GDP of region i from 2011 to 2021.

#### 3.4.4 Spatial autocorrelation test

The existence of spatial links is the basis of spatial econometric analysis, which requires a spatial matrix to calculate the Moran index and determine whether there are spatial links between regions based on the Z value of the Moran index [53]. In this study, a geographic distance matrix is used to calculate the Moran index between the regions each year.

When the Moran's index test is significant, it indicates that the variable has spatial correlation. Otherwise, the spatial correlation is not statistically significant. The value of Moran's index is between −1 and 1. A Moran's index greater than 0 indicates a positive spatial correlation in the region, and vice versa. The larger the absolute value of the Moran index, the stronger its spatial correlation. The Moran index is divided into global Moran index and local Moran index. The former is used to examine the clustering of the entire spatial sequence, while the latter is used to examine the spatial clustering within a local region. The global Moran's I is calculated as follows:

$$Gobal\;Moran^{\prime }s\;I=\frac{n\sum_{i=1}^{n}\sum_{j=1}^{n}w_{ij}\left(x_{i}-\bar{x}\right)\left(x_{j}-\bar{x}\right)}{\sum_{i=1}^{n}\sum_{j=1}^{n}w_{ij}\sum_{i=1}^{n}{\left(x_{i}-\bar{x}\right)}^{2}}$$

The local Moran's I is calculated as follows:

$$Local\;Moran^{\prime }s\;I=\frac{n\left(x_{i}-\bar{x}\right)\sum_{j=1}^{n}\left(x_{j}-\bar{x}\right)}{\sum_{i=1}^{n}{\left(x_{i}-\bar{x}\right)}^{2}}$$

#### 3.4.5 Spatial panel model

The selection of spatial models is on the basis of the results of the LM test, the LR test, and the Hausmann test with reference to Ref. [54]. The results of the tests are shown in section 4.4.3. On the basis of the tests, the spatial Durbin model (SDM) in equation (3) is employed to analyze the spatial spillover effect of digital economy on rural revitalization. The spatial Durbin model can not only be used to examine the impact of the digital economy in neighboring areas on local rural revitalization, but also includes the impact of rural revitalization in neighboring areas on local rural revitalization, thereby analyzing the spatial effects between two regions. The results of the spatial autoregressive model are presented in order to ensure the robustness of the results.

(3) $${Rural}_{it}=\delta_{0}+\rho W{Rural}_{it}+\delta_{1}{\text{Digit}}_{it}+\delta_{2}W{\text{Digit}}_{it}+\delta_{3}Z_{i,t}+\delta_{4}WZ_{i,t}+\sigma_{i}+\mu_{it}$$

Where, ρ is the spatial autoregressive coefficient, W is the spatial weight matrix, δ is the coefficient to be estimated, $\sigma_{i}$ denotes the individual fixed effect of city i that does not change over time [55,50].

### 3.5 Robustness test

#### 3.5.1 Removal of outliers

In order to alleviate the estimation bias caused by outliers, the comprehensive index of digital economy and rural revitalization underwent a second estimation after contracting by 1%.

#### 3.5.2 Control of city-specific fixed effects

There are great differences in geographic location, resource endowment and policy support across regions, and failure to mitigate the effects of these underlying factors may lead to endogeneity problems, which can lead to estimation bias and pseudo-correlation. Therefore, the estimation is conducted by controlling for individual fixed effects.

#### 3.5.3 Replacement of core explanatory variables

Despite constructing digital economy indicators based on multiple dimensions, there may still be information omissions that can lead to endogeneity problems. Referring to Ref. [52], the number of listed companies in the digital industry of each city is included in the digital economy index system, and the entropy method is used to obtain a new digital economy development index to replace the previous digital economy development index for analysis. The number of listed companies in digital industry in each city is obtained according to the CNRDS database of listed companies' governance basis database.

#### 3.5.4 Impact of COVID-19

Whether the COVID-19 that broke out at the end of 2019 and the subsequent government measures to prevent and control COVID-19 will have an impact on rural revitalization should be included in this study. Therefore, we exclude the data for 2020 & 2021 for estimation. The results excluding 2020 and 2021 are compared with those for the two years included. Then the impact of the COVID-19 epidemic is discussed.

#### 3.5.5 Instrumental variables approach to endogeneity

For the endogeneity problem, instrumental variables can effectively mitigate the effects brought by two-way causality. Referring to Ref. [53], the number of fixed telephone sets in the sample cities during 1984 was taken as the instrumental variable of digital economy. As a traditional communication technology, the use of the fixed-line telephone fulfills the exogenous requirements of the instrumental variable, as it influences the subsequent development of the digital economy and is gradually replaced by new communication tools. The number of fixed telephone sets during the 1984 period is only cross-sectional data, and in order to match it with the panel data, an instrumental variable for the digital economy is constructed by introducing a variable that varies over time with reference to the methodology of [54]. Specifically, an interaction term is constructed between the number of Internet users in the province in that year and the number of telephones per 10,000 people in each city in 1984, lagged one period in time, as an instrumental variable for the digital economy in that year.

### 3.6 Heterogeneity analysis

Differences in regional resource endowments, geographic locations and policy preferences can lead to significant regional heterogeneity in the level of development of the digital economy and rural revitalization in various regions. Therefore, the effect of the digital economy on rural revitalization may have the same regional heterogeneity, which requires further in-depth discussion of its effect. Therefore, we analyze the heterogeneity of the PRD, the East Wing, the West Wing and the mountainous regions of Guangdong Province.

## 4 Results

### 4.1 Descriptive statistics

Table 3 shows the descriptive statistics of the variables. The mean of the Rural Revitalization Composite index is 0.2103, while the maximum and minimum value is 0.6042 and 0.0963 respectively, indicating there is great difference between regions. The average value of the Digital Economy Development Level Index (Digit) is 0.1319, while the maximum and minimum value is 0.6921 and 0.0060, indicating a “spindle-shaped” distribution of the level of digital economy development among the regions. The level of agriculture, forestry and water expenditures, the level of urbanization, and the investment in fixed assets in agriculture, forestry, animal husbandry and fishery also have obvious differences between the regions.

**Table 3:** Descriptive statistics of the variables.

Variables	Sample	Mean	Std. Dev.	Max	Min
Rural Revitalization Development Index	220	0.2103	0.1086	0.6042	0.0963
Digital Economy Development Index	220	0.1319	0.1298	0.6921	0.0060
Level of agriculture, forestry and water expenditures	220	0.0461	0.0408	0.4714	0.0103
Level of urbanization	220	0.6137	0.1834	0.9521	0.3592
Investment in fixed assets in agriculture, forestry, animal husbandry and fishery	220	0.0201	0.0206	0.0840	0.0002

### 4.2 Evolutionary trend and spatial distribution pattern of rural revitalization in Guangdong Province

With reference to the Guangdong Statistical Yearbook, Guangdong Province is categorized into four regions: the Pearl River Delta (PRD), the East Wing, the West Wing and the Mountainous Region. Among them, the PRD includes Guangzhou, Zhuhai, Foshan, Jiangmen, Dongguan, Zhongshan, Huizhou and Zhaoqing; the East Wing includes Shantou, Shanwei, Chaozhou and Jieyang; the West Wing includes Zhanjiang, Maoming and Yangjiang; and the Mountainous Region includes Shaoguan, Heyuan, Meizhou, Qingyuan and Yunfu. The index of digital economy and rural revitalization is divided into high-level (greater than 0.4), medium-level (between 0.2 and 0.4) and low-level (less than 0.2) using the equal spacing separation method, and the spatial distribution of the two is plotted using ArcGIS.

**Fig. 1:**
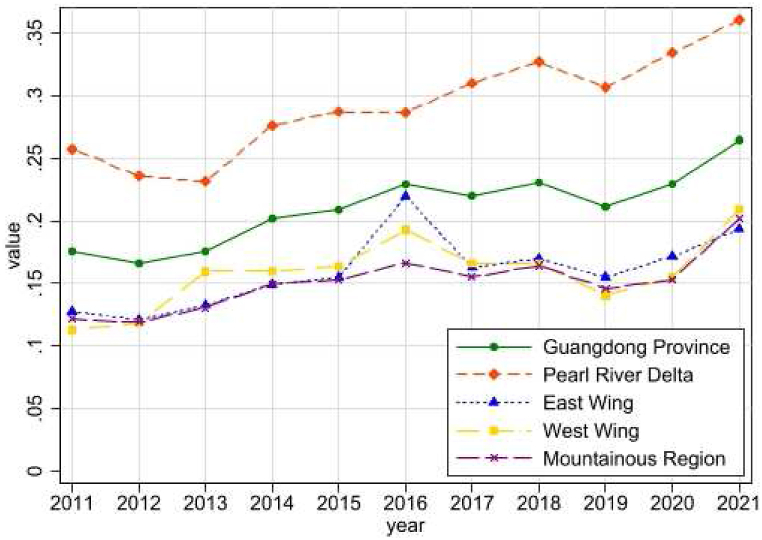
Revolutionary trend of rural revitalization in Guangdong Province.

Fig. 1 shows the revolutionary trend of rural revitalization in Guangdong Province. Overall, the level of rural revitalization in Guangdong Province has shown a trend of fluctuating increase during 2011–2021, and the overall growth rate has remained stable with only a short period of insignificant drop-off. The level of rural revitalization in all four regions is on an upward trend, with only the Pearl River Delta (PRD) region standing out. It started at about 0.26 in 2011 and ended at about 0.36 in 2021 in the PRD region, where the rural revitalization is greater than that of the other three regions.

With the exception of 2012, 2013, and 2019, when there was a short fall, the level of development of rural revitalization in the PRD region was well-founded and had generally shown a sustained and rapid upward trend. The rural revitalization comprehensive indexes of the East Wing Region, West Wing Region and Mountainous Region had a weaker base, and in 2011 they were all lower than the Guangdong Provincial Rural Revitalization Comprehensive Index. The Rural Revitalization Comprehensive Indexes of both the East Wing region and the West Wing region showed some fluctuations, with the East Wing being the one that experiences large fluctuations in 2015 and 2016, while the West Wing experienced a steady increase in 2011–2016 and a yearly decrease in 2016–2019. Similar to the overall trend in Guangdong Province, the overall rural revitalization composite index for mountainous regions had maintained a sustained upward trend overall, except for a brief decline in 2017 and 2019, with a larger increase in 2019–2021, which ranked as the second highest growth rate of the four regions.

**Fig. 2:**
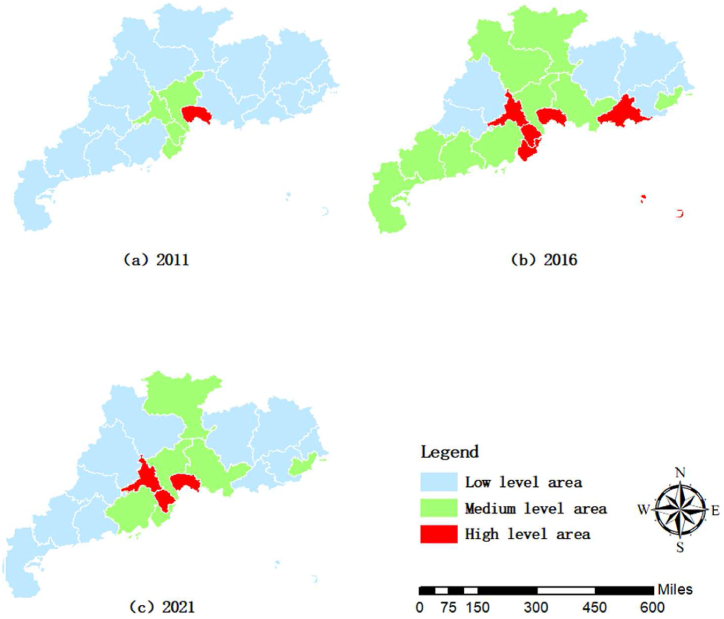
Spatial distribution of rural revitalization in Guangdong Province.

Fig. 2 shows the spatial distribution of rural revitalization in Guangdong Province by intercepting the data in 2011 (Fig. 2 (a)), 2016 (Fig. 2 (b)) and 2021 (Fig. 2 (c)). It can be seen that high-level areas in rural revitalization are concentrated in the PRD region. Only Dongguan was in the high-level area in 2011 and 2016. Guangzhou, Foshan, Zhongshan and Zhuhai were medium-level areas in 2011, and Huizhou and Shanwei became medium-level zones in 2016. In 2021, Dongguan, Shenzhen, Foshan and Zhongshan were the high-level area of rural revitalization. Compared with 2016, the cities of Foshan and Zhongshan in the PRD region were upgraded from a medium-level region to a high-level region. The medium-level area was distributed in eleven cities: Guangzhou, Zhuhai, Shaoguan, Shantou, Huizhou, Zhaoqing, Jiangmen, Yunfu, Yangjiang, Maoming and Zhanjiang. Only six cities were in the-low level area: Qingyuan, Heyuan, Meizhou, Shanwei, Jieyang, and Chaozhou. In 2021, the city of Shaoguan in the mountainous region and many cities in the western region were upgraded from low-level to medium-level regions, while in the eastern region, Shanwei was downgraded from a medium-level region to a low-level one, and Shantou was upgraded from low-level to medium-level.

### 4.3 Evolutionary trend and spatial distribution pattern of digital economy in Guangdong Province

**Fig. 3:**
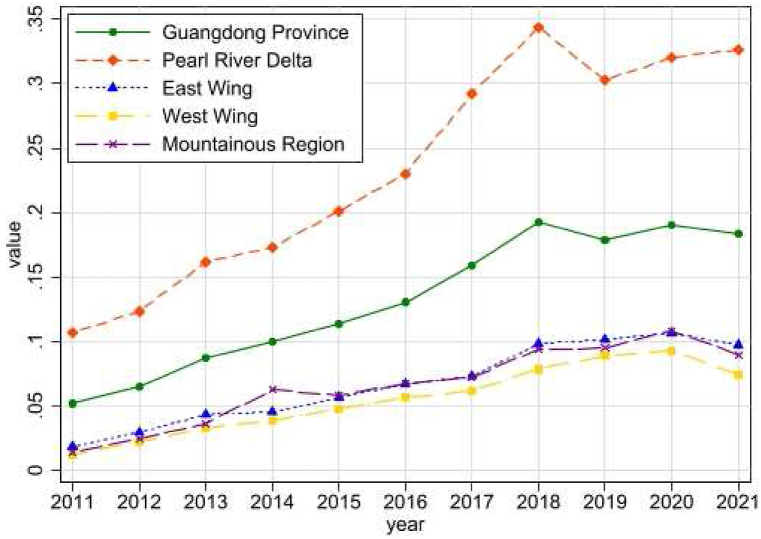
Revolutionary trend of digital economy in Guangdong Province.

Fig. 3 shows the revolutionary trend of digital economy in Guangdong Province. The Digital Economy Index is generally on an upward trend, except for a drop in 2019. During the study period, the level of the digital economy in the Pearl River Delta (PRD) was ahead of the other three regions and the overall level of Guangdong Province, taking a dominant position in the process of the development of the digital economy in Guangdong Province. The digital economy development levels of the East Wing, the West Wing and the Mountainous Region were roughly the same.

**Fig. 4:**
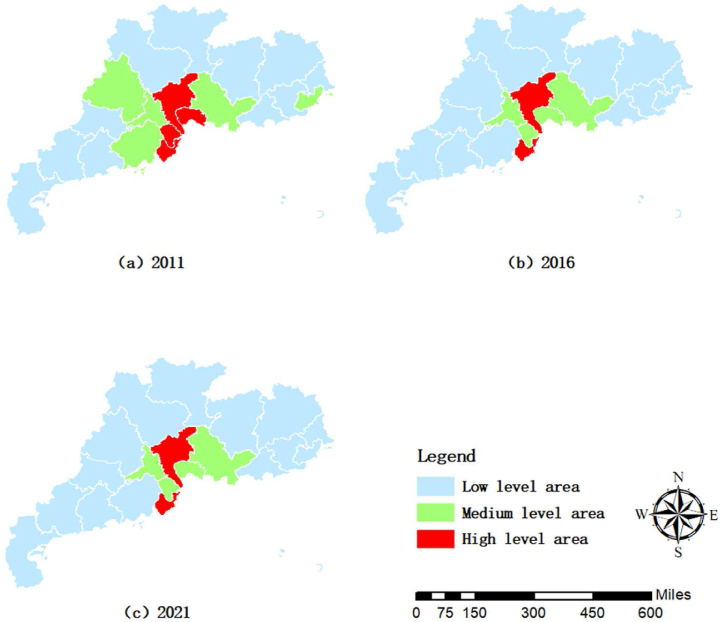
Spatial distribution of digital economy in Guangdong Province.

Fig. 4 displays the spatial distribution of digital economy in Guangdong Province. Fig. 4 (a) shows that in 2011 all the regions were in low-level digital economy areas, and there were small differences between the Pearl River Delta (PRD), the East Wing, the West Wing and the mountainous regions. Fig. 4 (b) shows that in 2016, the high-level digital economy area was concentrated in Guangzhou and Zhuhai in the PRD region, and the medium-level digital economy area was concentrated in Dongguan and Zhongshan in the PRD region. Compared with 2011, the four cities were in more advanced position in terms of digital economy development. Fig. 4 (c) shows that in 2021, the high level of digital economy is concentrated in Guangzhou and Zhuhai in the PRD region, and the medium level of digital economy is concentrated in Dongguan, Zhongshan, Huizhou and Foshan in the PRD region. Compared with 2016, there are two more cities, Huizhou and Foshan, in the ranks of medium-high level of digital economy development. Overall, digital economy development in the Pearl River Delta region shows a rising and radiating trend, while the other three regions are lagging behind relatively.

### 4.4 Model regression results

#### 4.4.1 Benchmark model results

Table 4 reports the results of benchmark model. The results in columns (1) and (2) show that the estimated coefficients of the core explanatory variable. The coefficient of Digital Economy Development Index (Digit) is equal to 0.3537 (without control variables), which is significantly positive, indicating that there is a positive impact of digital economy on rural revitalization. Further, after controlling for related variables, the coefficient is still significantly positive (0.2995), validating Hypothesis 1 of this study. The results in column (2) show that there is a positive effect of expenditures on agriculture, forestry and water conservancy facilities (level_ATW) and level of urbanization (Urban) on rural revitalization, and the coefficients are 0.1218 and 0.3295. Regional government expenditures on agriculture, forestry and water conservancy facilities refers to the financial investment made by the government, farmers, enterprises, or other institutions to promote agriculture, forestry, and water resource management. As an important input in the rural industry, it tends to increase along with rural development. The level of urbanization (Urban) has a significant positive impact on rural revitalization, which is consistent with the research findings of [56,57].

**Table 4:** Results of benchmark model.

Variables	(1)	(2)
	Rural	Rural
Digital Economy Development Index	0.3537***	0.2995***
	(0.0795)	(0.0766)
Expenditures on agriculture, forestry and water conservancy facilities		0.1218***
		(0.0432)
Level of urbanization		0.3295***
		(0.000945)
Investment in fixed assets in agriculture, forestry, animal husbandry and fishery		0.1986
		(0.2179)
Constants	0.164***	−0.0298
	(0.0185)	(0.0462)
*N*	220	220
*R*2	0.3476	0.3739

Note: *, **, *** indicate significance at the level of 0.1, 0.05 and 0.01 respectively; Standard errors in parentheses.

The impact of government financial input (Capi) on rural revitalization is not significant. The main reason for this is that the population of Guangdong Province is concentrated in the Pearl River Delta region as well as in urban centers [58], with a relative scarcity of labor in the countryside, leading to a reduction in the marginal output and return on investment, which could be explained by the Heckscher-Ohlin Theorem [59].

#### 4.4.2 Threshold model results

After conducting 1000 bootstrap samples, the threshold test results are shown in Table 5, which indicates that it passed the single threshold and double threshold tests, but did not pass the triple threshold test. Therefore, the double threshold model is selected in this study.

**Table 5:** Threshold test results.

threshold	F-statistics	P-value	10% significance level	5% significance level	1% significance level
Single threshold	37.40	0.0040***	17.4810	20.7267	30.8445
Double threshold	33.02	0.0090***	17.6713	21.8516	30.7194
Triple threshold	21.25	0.7730	68.2082	82.4975	100.2717

The results of the threshold model in Table 6 show that in the regions with a low level of digital economic development($Digit\times I\left(Digit\leq q_{1}\right)$), the existence of digital divide leads to a negative impact of the digital economy on rural revitalization [60], where the coefficient is −0.1847; while in the regions with a mediu*m (*$\text{Digit}\times I\left(q_{1}＜\text{Digit}\leq q_{2}\right)$ *or* high level ($Digit\times I\left(\text{Digit}＞q_{2}\right)$ of digital economic development, the digital economy has a significant positive effect on rural revitalization, where the coefficients are 0.2193 and 0.3835. Thus, the effect of digital economy on rural revitalization has non-linear characteristics, thus verifying hypothesis 2.

**Table 6:** Results of threshold model.

Threshold Value	$q_{1}$	0.1652
	$q_{2}$	0.3605
Variables		Rural
$\text{Digit}\times I\left(\text{Digit}\leq q_{1}\right)$		−0.1847**
		(0.0732)
$\text{Digit}\times I\left(q_{1}＜\text{Digit}\leq q_{2}\right)$		0.2193***
		(0.0352)
$\text{Digit}\times I\left(\text{Digit}＞q_{2}\right)$		0.3835***
		(0.0336)
Control relevant variables		YES
N		220
R2		0.5415

Note: *, **, *** indicate significance at the level of 0.1, 0.05 and 0.01 respectively; Standard errors in parentheses.

#### 4.4.3 Spatial model results

**Fig. 5:**
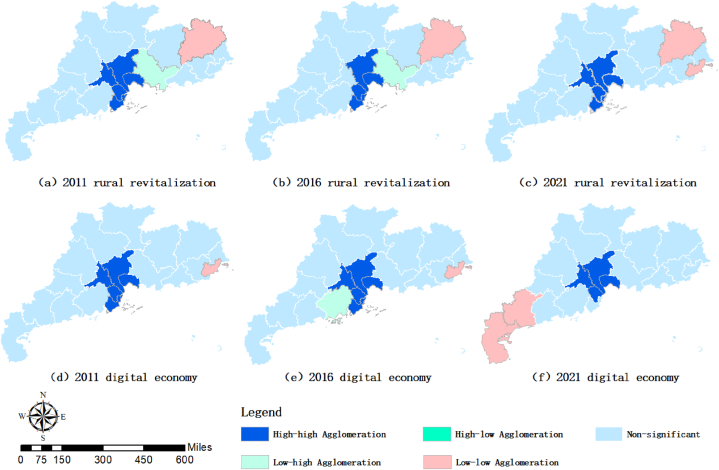
The spatial pattern of the development of digital economy and rural revitalization.

The results of Moran's index between cities under each year by using a geographic distance matrix are reported in Table 7, which shows that both the digital economy and rural revitalization Moran's I passed the 1% significance level test from 2011 to 2021, indicating that both have spatial clustering, i.e., there is a spatial connection. In order to observe the spatial connection between digital economy and rural revitalization more intuitively, this study uses ArcGIS software to map the spatial pattern of digital economy and rural revitalization. Fig. 5(a–c) shows the spatial pattern of rural revitalization in 2011, 2016 and 2021. Figure (d–f) shows the spatial pattern of the development of digital economy. Both digital economy and rural revitalization have high-high agglomeration in the PRD region, which indicates that there is a significant spatial correlation between the development of digital economy and rural revitalization in the PRD region. Rural revitalization has the spatial phenomenon of low-low agglomeration in the East Wing, while low-high agglomeration exists around the PRD region.

**Table 7:** The Moran‘I index of Digital Economy Development and Rural Revitalization Development.

year	Digital Economy Development		Rural Revitalization	
	Moran'I	Z-value	Moran'I	Z-value
2011	0.2384***	3.5729	0.1677***	3.1550
2012	0.2389***	3.5816	0.1997***	3.5916
2013	0.2052***	3.2557	0.1815***	3.2927
2014	0.1967***	3.1294	0.2030***	3.6437
2015	0.2193***	3.4278	0.1857***	3.3527
2016	0.2140***	3.3945	0.1425***	2.5104
2017	0.2519***	3.8080	0.2084***	3.2763
2018	0.2467***	3.7712	0.2295***	3.5793
2019	0.1675***	2.9932	0.2142***	3.5349
2020	0.1582***	2.8892	0.1817***	2.9994
2021	0.1649***	2.9578	0.2096***	3.4056

Note: *, **, *** indicate significance at the level of 0.1, 0.05 and 0.01 respectively.

With reference to Ref. [55], the spatial model is selected by LM test, LR test, and Hausman test, combining the test results, which are displayed in Table 8. The LM test showed that there were spatial lag effects as well as spatial error effects, therefore, the spatial Durbin model should be selected. The Hausman test showed that the fixed effects model was superior to the random effects model. To determine whether to choose individual fixed effects or time fixed effects or two-way fixed effects, a Hausmann test was conducted, which indicated that the individual fixed effects model was appropriate. The LR test showed that the SDM model could not degenerate into a spatial autoregressive model (SAR) or a spatial error model (SEM). Therefore, the spatial Durbin model with individual fixed effects was chosen for this study.

**Table 8:** Results of spatial model selecting test.

Spatial model test		Statistics	P-value
LM Test	LM-ERR	18.241 ***	0.000
	Robust LM-ERR	12.919 **	0.000
	LM-LAG	6.133	0.013
	Robust LM-LAG	0.811	0.368
Hausman Test	FE vs RE	28.33***	0.001
	Ind vs Both	5.46	0.244
	Time vs Both	13.01**	0.011
	Ind vs Time	9.14*	0.058
LR Test	SAR vs. SDM	22.04***	0.000
	SEM vs. SDM	36.79***	0.000

Note: *, **, *** indicate significance at the level of 0.1, 0.05 and 0.01 respectively.

Table 9 reports the results of the SAR and SDM models with different matrices. Columns (4)–(6) report the results of the spatial Durbin model. The results in (5) show that under the geographic distance weight matrix, the digital economy significantly promotes local rural revitalization spatially, and there is a positive spatial spillover effect on rural revitalization in neighboring regions, and this result still holds after replaced with the economic distance matrix and the adjacency matrix. In addition, rural revitalization in the region also has spatial spillover effects on neighboring regions, which is proven by the significant results of the spatial autoregressive model. Thus, hypothesis 3 has been verified.

**Table 9:** The results of the SAR and SDM models.

Spatial Model	Spatial autoregressive model with individual fixed effect			Spatial Durbin model with individual fixed effect		
Spatial Matrix	Economic matrix	Geographical distance	Adjacency matrix	Economic matrix	Geographical distance	Adjacency matrix
Variables	(1)	(2)	(3)	(4)	(5)	(6)
	Rural	Rural	Rural	Rural	Rural	Rural
Digit	0.1973***	0.2118***	0.2442***	0.1204*	0.1291**	0.0947*
	(0.0721)	(0.0764)	(0.0793)	(0.0641)	(0.0551)	(0.0546)
W×Digit				0.1388*	0.1217**	0.2050***
				(0.0716)	(0.0522)	(0.0769)
W×Rural	0.5362***	0.4489***	0.3420***	0.3958***	0.3501***	0.2549***
	(0.0643)	(0.0613)	(0.0686)	(0.0650)	(0.0522)	(0.0636)
Direct effect	0.2109***	0.2254***	0.2563***	0.1350**	0.1451**	0.1148**
	(0.0766)	(0.0810)	(0.0825)	(0.0657)	(0.0566)	(0.0535)
Indirect effect	0.2140***	0.1585***	0.1143***	0.2977***	0.2426***	0.2865***
	(0.0748)	(0.0546)	(0.0361)	(0.1006)	(0.0675)	(0.0838)
Total effect	0.4248***	0.3839***	0.3706***	0.4327***	0.3876***	0.4012***
	(0.1430)	(0.1288)	(0.1088)	(0.1229)	(0.0926)	(0.0815)
Control relevant variables	YES	YES	YES	YES	YES	YES
*N*	220	220	220	220	220	220
*R*2	0.4371	0.4298	0.4189	0.4908	0.4753	0.4742

Note: *, **, *** indicate significance at the level of 0.1, 0.05 and 0.01 respectively; Standard errors in parentheses.

### 4.5 Robustness test results

#### 4.5.1 Removal of outliers

Table 10 reports the results of the robustness tests. Columns (1) and (2) report the results of the robustness test excluding outliers, which show that the positive effect of digital economy on rural revitalization is still significantly positive, and this conclusion is still robust after controlling for relevant factors, which further validates the research hypothesis 1.

**Table 10:** Results of robust test.

Robust test	Excluding outliers		Controlling relevant fixed effect		Replacing Digital Economy Development Index	
Variables	(1)	(2)	(3)	(4)	(5)	(6)
	Rural	Rural	Rural	Rural	Rural	Rural
Digital Economy Development Index	0.358***	0.3104***	0.3420***	0.3078***	0.2676***	0.2005***
	(0.0790)	(0.0794)	(0.0759)	(0.0797)	(0.0900)	(0.0698)
Level of agriculture, forestry and water expenditures		−0.2039**		−0.1170**		−0.1104**
		(0.0821)		(0.0415)		(0.0546)
Level of urbanization		0.3075***		0.3085**		0.3999***
		(0.0898)		(0.1386)		(0.0829)
Investment in fixed assets in agriculture, forestry, animal husbandry and fishery		0.2184		0.1758		0.0191
		(0.2297)		(0.2278)		(0.2103)
Constants	0.1634***	−0.0144	0.1651***	−0.0178	0.1795***	−0.0534
	(0.0186)	(0.0430)	(0.0100)	(0.0793)	(0.0202)	(0.0413)
*N*	212	212	220	220	220	220
*R*2	0.3198	0.3414	0.3476	0.3741	0.2255	0.2919

Note: *, **, *** indicate significance at the level of 0.1, 0.05 and 0.01 respectively; Standard errors in parentheses.

#### 4.5.2 Control of city-specific fixed effects

The results in columns (3) and (4) of Table 10 show that the impact of digital economy on rural revitalization remains significantly positive after controlling for fixed effects at the individual level, proving the positive effect of digital economy on rural revitalization in Guangdong Province.

#### 4.5.3 Replacement of core explanatory variables

The results in columns (5) and (6) of Table 10 show that the impact of the digital economy on rural revitalization remains significantly positive after replacing the digital economy index, a result that remains robust to the inclusion of control variables.

#### 4.5.4 The impact of COVID-19

Table 11 shows the comparison between the results with or without the year 2020 and 2021. Column (1) and (2) present the results with the two years excluded and included. As can be seen, there is a slight difference (from 0.285 to 0.2995) in the coefficients of the digital economy between two results, which implies that the outbreak of COVID-19 and the anti-epidemic measures had little impact on rural revitalization.

**Table 11:** The impact of COVID-19.

	The impact of COVID-19	
	(1)	(2)
	Rural	Rural
Digit	0.285***	0.2995***
	(0.0700)	(0.0766)
level_ATW	−0.141***	0.1218***
	(0.0396)	(0.0432)
Urban	0.283***	0.3295***
	(0.0990)	(0.000945)
Capi	0.120	0.1986
	(0.203)	(0.2179)
cons	0.0000205	−0.0298
	(0.0471)	(0.0462)
*N*	180	220
*R*2	0.3145	0.3739

Note: *** indicate significance at the level of 0.01; Standard errors in parentheses.

#### 4.5.5 Endogeneity

Table 12 reports the results of the estimation using the instrumental variables approach. The estimation results in columns (1) and (2) show that the digital economy still has a significant positive effect on rural revitalization after the introduction of instrumental variables. Tests using the Kleibergen-Paap rk LM statistic show that it passes the test of significance at the 1% level, which indicates that there is no over-identification of instrumental variables. A test using the Kleibergen-Paap rk Wald F statistic shows that the value of this F statistic is greater than the critical value at the 10% level in the test for weak instrumental variables, which suggests that there is no problem of weak instrumental variables. The above analysis justifies the selection of instrumental variables in this paper. The validity of the instrumental variables is further verified using the Kleibergen-Paap rk LM statistic and the Wald F statistic, and the positive impact of the digital economy on rural revitalization is also verified.

**Table 12:** Results of the estimation using the instrumental variables.

Variables	(1)	(2)
	Rural	Rural
Digit	0.4263***	0.3838***
	(0.0467)	(0.0548)
Control relevant variables	NO	YES
Kleibergen-Paap rk LM statistics	20.805	24.291
	[0.0000]	[0.0000]
Kleibergen-Paap rk Wald F statistics	53.441	62.141
	{16.38}	{16.38}
N	220	220
*R*2	0.1327	0.1251

Note: *, **, *** indicate significance at the level of 0.1, 0.05 and 0.01 respectively; Standard errors in parentheses; P-values in square brackets; Critical values at the 10% level for the Stock-Yogo weak identification test in curly brackets.

### 4.6 Results of heterogeneity analysis

The results of the descriptive statistics by region in Table 13 show that the level of development of the digital economy as well as rural revitalization in the PRD Region is far ahead of the other three regions. Furthermore, the East Wing, West Wing and Mountainous Region have similar levels of digital economy and rural revitalization development, with less fluctuation.

**Table 13:** Descriptive statistics by region.

(a) Digital Economy Development Index					
Region	Sample	Mean	Std. Dev.	Max	Min
Pearl River Delta	88	0.2347	0.1521	0.6921	0.0369
East Wing	44	0.0669	0.0328	0.1294	0.0060
West Wing	33	0.0550	0.0267	0.0994	0.0061
Mountainous region	55	0.0655	0.0320	0.1252	0.0092
(b) Rural Revitalization Development Index					
Region	Sample	Mean	Std. Dev.	Max	Min
Pearl River Delta	88	0.2920	0.1271	0.6042	0.1010
East Wing	44	0.1598	0.0470	0.3862	0.1008
West Wing	33	0.1585	0.0317	0.2133	0.0996
Mountainous region	55	0.1509	0.0351	0.2727	0.0963

Table 14 reports the results of the regional heterogeneity regression analysis. The estimation results in columns (1)–(4) show that the digital economy has a significant effect on rural revitalization in each region, but there is regional heterogeneity. Specifically, the positive effect in the PRD region is larger than that in the mountainous regions and smaller than that in the East and West wings, which may be due to the higher resource endowment of the PRD region and the non-linear effect of the digital economy, resulting in a smaller impact of the digital economy on its rural revitalization than that of the East and West wings. The East and West Wings' locational advantages over the mountainous regions enable them to give full play to the impact of the digital economy on rural revitalization. The advantages of rapid logistics turnover in the East and West wings fully ensure the healthy circulation of the industrial logistics chain and capital chain, and steadily promote the development of rural revitalization. The mountainous areas, however, do not have these advantages, and the existence of the digital divide will further slowdown the promotion of the digital economy on rural revitalization.

**Table 14:** Results of the regional heterogeneity regression.

Region	Pearl River Delta	East Wing	West Wing	Mountainous Region
Variable	Rural	Rural	Rural	Rural
Digital Economy Development Index	0.3141***	0.7215**	0.5467***	0.2238***
	(0.0846)	(0.3118)	(0.0477)	(0.0842)
Level of agriculture, forestry and water expenditures	−1.4221***	−0.0888	−0.5516***	0.2408
	(0.4404)	(0.1440)	(0.2109)	(0.2429)
Level of urbanization	0.4892***	0.1150**	0.1960***	0.3078***
	(0.1641)	(0.0572)	(0.0421)	(0.1084)
Investment in fixed assets in agriculture, forestry, animal husbandry and fishery	−0.1673	1.0079*	0.5273***	0.3373***
	(0. 2069)	(0.5819)	(0.1505)	(0.1281)
Constants	−0.0981	0.0234	0.0519**	−0.0284
	(0.1083)	(0.0345)	(0.0212)	(0.0425)
*N*	88	44	33	55
*R*2	0.5887	0.1780	0.2885	0.2927

Note: *, **, *** indicate significance at the level of 0.1, 0.05 and 0.01 respectively; Standard errors in parentheses.

## 5 Discussions

This paper measures the rural revitalization and digital economy in Guangdong province during 2011–2021 and explores the effect of digital economy on rural revitalization. The spatial panel model and the threshold model are employed to analyze the spatial effects and marginal effects of digital economy on rural revitalization. The findings of this study help to understand the value of developing digital economy to improve rural revitalization.

### 5.1 Digital economy's effect on the rural revitalization

The findings of the study revealed that digital economy had significant positive effect on the rural revitalization in Guangdong province, verifying Hypothesis 1. Digital technologies have great potential to support rural areas in improving economic, social and environmental sustainability [61]. [37] The digital economy can play a role in all aspects of rural revitalization, including industrial prosperity, ecological livability, rural civilization, effective governance, and affluent living. Hou et al. (2023) analyzed panel data from 31 provinces in China from 2013 to 2021 using the mediated effects model and the spatial Durbin model, and concluded that the digital economy can promote rural revitalization by facilitating industrial structural transformation and rural entrepreneurship, and that the digital economy generates spillover effects that lead to rural revitalization in neighboring areas [39]. This study is based on data from 20 prefectural-level cities in Guangdong Province, and draws similar conclusions, suggesting that the digital economy in Guangdong Province is equally instrumental in promoting rural revitalization. Although Guangdong Province is at the forefront of China in terms of both economic development and the development of the digital economy, its development is highly uneven, with large differences in the development of the Pearl River Delta (PRD), the East and West Wings, and the Mountainous Regions, similar to the overall situation in China, where there are large differences in the development of the three regions of the East, Central and West. It is therefore not surprising that similar conclusions can be drawn from the data for Guangdong Province. According to this, it is necessary to enhance the digital economy in rural areas in order to achieve the goal of rural revitalization. The mediation effect analysis results given in the current study [39] reveal that the digital economy affects the intermediary variables of rural entrepreneurship and transformation of industrial structure, thus affecting the rural revitalization.

### 5.2 Heterogeneity and non-linear characteristics among regions

The results of the regression of regional heterogeneity of the digital economy on rural revitalization show that the positive effect in the PRD region is larger than in the mountainous regions, but smaller than in the east and West Wings. The findings of the threshold model show that the impact of the digital economy on rural revitalization shows nonlinearity, which verifies research hypothesis 2. Specifically, the impact of the digital economy on rural revitalization is related to the level of digital economic development in each region. The regional heterogeneity and non-linear character of the impact of the digital economy has been confirmed in other studies, such as the impact of the digital economy on urban carbon emissions [62] and the impact of the digital economy on high-quality green development [63].

The impact of digital economy shows non-linearity indicates that the marginal impact of digital economy on rural revitalization is changing. In this paper, the best development of digital economy is in the PRD region, followed by the east and West Wings, and finally the mountainous areas. In the PRD region and east and West Wings where the digital economic development is at medium to high level, the digital economy tends to have positive effect on the rural revitalization. But in mountainous areas where the digital economy is lagging behind, the opposite is true. This indicates that the marginal impact of the digital economy shows a trend of increasing first and then diminishing. Digitization and digital technologies as important factors of production in modern economies have both an increasing marginal impact due to the characteristics of network externalities and a diminishing marginal impact similar to that of traditional factors at a certain level of development.

Compare the east and West Wings with the mountainous areas and we can see the advantages of east and West Wings that they have better infrastructure, especially port resources. The east and West Wings are on the coast and are more accessible than the mountains, and it is not surprising that the digital economy has had a greater impact on these regions than on the mountains. While the economy of the PRD region is already quite developed, the aggregation effect brought about by the digital economy is obvious, and it will also bring about environmental and transportation problems to these areas, thus greatly reducing the role of the digital economy in rural revitalization.

Another factor that affects the role of the digital economy in rural revitalization is the information literacy and digital skills of local people, which is one of the major reasons for the digital divide [28,64]. Information literacy and digital skills are the abilities to obtain the required information and services and to convey messages through appropriate digital devices [65]. Information literacy is the foundation of lifelong learning in the Information Age and the ability to utilize information and communication technology to achieve specific personal and professional goals, which can be divided into two parts: understanding information contents and competency in using ICT. Information literacy is related to the digital skills to grasp information content, to identify when information is needed, and effectively locate, evaluate, and use the required information to effectively respond to media. For example, before selling on an e-commerce platform, you must be able to create a user account and register on the platform. It is necessary to take photos and videos of the products and upload them to the platform, all of which require mastery of information and communication technology and the manipulation of complex information and communication applications [65]. Thanks to the development of information technology in China, the vast majority of Chinese people, including rural residents, have access to mobile phones for internet access, but this does not mean the disappearance of the digital divide, which is now mainly caused by the differences in the information literacy and digital skills among different population groups. The rural population is dominated by the elderly and women with little information literacy and digital skill because young people migrate to the cities [58,66]. Promoting information literacy and digital skills among rural populations has therefore become an important strategy for rural revitalization.

### 5.3 Spatial spillover effects on rural revitalization

The findings in this study reveal that the digital economy has a spatial spillover effect on rural revitalization, which is consistent with the current literature [37,39,67]and verifies Hypothesis 3. Hou et al. (2023) drew the conclusion that there was the spillover effect of the digital economy on rural revitalization. Meng (2022) used the spatial Durbin model to measure the impact of digital financial inclusion on the development of rural revitalization. It was found that the digital inclusive finance can help the revitalization and there exists spillover effect [41]. According to the results based on the Durbin spatial lag model applied to Japanese regional data, this is mainly because investment in informatization technology and informatization diffusion help to break the economic spatial constraints caused by distance, thus generating interregional spillover effects [68]. The direct impact of informatization on the local area and the indirect impact on the surrounding area are positive. This proves the existence of network externalities, leading to incremental returns to scale. The wide diffusion of information technology plays an important role in this process, and the rapid accumulation and penetration of information resources reinforces the spillover effects of investment in informatization.

### 5.4 Impact of COVID-19

The findings in this study show that the COVID-19 epidemic had little impact on rural revitalization in Guangdong Province. Apparently, the impact of the epidemic on rural economic and cultural development, rural residents' lives, rural governance and ecological environment is what our study failed to reveal. Existing researches show that the pandemic had a huge impact in many ways. As known to all, nationwide/region-based lockdown strategies were adopted to contain the outbreak of the epidemic by some of the COVID-19 affected countries. And this in turn resulted in restricted transportation [69] and improved air quality as a side effect [70]. Thus, it posed challenge on the development of logistics [71] and many households in rural area suffered from a loss of employment and a remarkable decrease in household income during the time of the COVID-19 spread [72]. The impact of the epidemic on agricultural production was much smaller compared to the impact on industrial production. In less populated rural areas, the virus did not spread as fast as in densely populated urban areas. As a result, agricultural production in Guangdong Province was not much affected during the epidemic. For example, according to the Guangdong Rural Statistical Yearbook, the output of grain from 2019 to 2021was 12.41 million tons, 12.676 million tons, 12.799 million tons, showing a growing trend. And the output of other crops as well as animal husbandry and fishery in Guangdong Province was growing too. Therefore, the development of the primary industry has not been greatly affected by the epidemic. Due to the adoption of digital technology, social governance and cultural development in rural areas were not significantly affected the epidemic. As mentioned earlier, the ecological environment has actually improved due to the decrease in personnel mobility. The biggest negative impact of lockdown should be on the off-farm income of rural residents, but because this is only a part of the rural revitalization index in this study, it is not fully reflected in our empirical results. In future research, the impact of the COVID-19 epidemic can be analyzed as an impact factor, which will lead to more interesting findings.

### 5.5 Policy implications

Based on the above analysis, the following recommendations are made. First, more policies should be formulated and implemented to promote the use of the digital economy and technology in rural areas, especially rural e-commerce platforms and inclusive finance. The Government shall guide the application of the digital economy and digital technology in the countryside so as to take advantage of the spillover effect of the digital economy to spread to more areas and achieve comprehensive rural revitalization. Second, it is recommended to upgrade the infrastructure in mountainous areas, especially the transportation capacity, in order to increase the marginal benefit of the digital economy for rural revitalization. Thirdly, it is strongly recommended to improve the information literacy and digital skills of the rural population so that the digital economy can play a greater role in rural revitalization. This can be done in two ways: on the one hand, by training existing rural residents in information literacy and digital skills, and on the other hand, by raising their incomes and improving the infrastructure in the countryside to attract the young people who are already digitally skilled and literate to return to their hometowns.

### 5.6 Limitations

We recognized several limitations of this study. First, due to the difficulty of data collection, the composite index of digital economy and rural revitalization is not comprising every aspect of the variables. Second, due to the unavailability of data, this study is unable to conduct empirical analysis on the mechanism of the impact of digital economy on rural revitalization. The mediation effect analysis is not carried out in this study. The index of rural revitalization in this study includes the variable industry structure measured by number of employees in the primary industry/number of employees in rural areas. With 21 variables already included in the Rural Revitalization Index of this paper, it is difficult to get other variables with available data for mediated effects analysis. According to the theoretical framework of this study, the use of digital technology brings about a reduction in search costs, replication costs, transportation costs, tracking costs and verification costs, and it can lead to an increase in the number of employees engaged in off-farm jobs, but these data are not available currently. Therefore, the absence of a mediation effects analysis is a limitation of this paper, which could be remedied in future research in this area.

## 6 Conclusions

This study analyzes the spatial and evolutionary pattern of rural revitalization and digital economy in Guangdong, China. The effect of digital economy on rural revitalization is analyzed using the spatial model and threshold model. The main conclusions are as follows: First, there is significant spatial differentiation in the digital economy and rural revitalization in Guangdong, China. Second, the digital economy has significant spatial spillover effect on the rural revitalization. Third, this effect is found out to have heterogeneous and non-linear characteristics. Overall, this study provides data-based evidence for the government to formulate policies to improve the rural revitalization.

## Ethics statement

Review and approval by an ethics committee was not needed for this study because there were no animal experiments, live vertebrates and higher invertebrates, human and behavioral studies involved in this study. For the same reason, informed consent was not required.

## CRediT authorship contribution statement

**Xueqin Deng:** Writing – review & editing, Writing – original draft, Validation, Supervision, Project administration, Funding acquisition, Formal analysis, Data curation. **Mingshan Huang:** Writing – original draft, Visualization, Software, Methodology, Data curation. **Rong Peng:** Writing – review & editing, Methodology, Funding acquisition, Conceptualization.

## Declaration of competing interest

The authors declare that they have no known competing financial interests or personal relationships that could have appeared to influence the work reported in this paper.
