# Supplementary material for: The impact of digital economy on rural revitalization: Evidence from Guangdong, China
Source: Heliyon. 2024 Mar 28;10(7):e28216. doi: 10.1016/j.heliyon.2024.e28216 (PMC11004695; doi:10.1016/j.heliyon.2024.e28216)
Supplement: Multimedia component 1 [file mmc1.docx]

Appendix

**Table S1 The ranges of rural revitalization classification**

| Year | Low-level area | Medium-level area | High-level area |
| --- | --- | --- | --- |
| 2011 | 0.096332 - 0.192274 | 0.192275 - 0.309516 | 0.309517 - 0.539398 |
| 2012 | 0.100824 - 0.166379 | 0.166380 - 0.289488 | 0.289489 - 0.478623 |
| 2013 | 0.108884 - 0.166113 | 0.166114 - 0.290618 | 0.290619 - 0.447199 |
| 2014 | 0.117578 - 0.211143 | 0.211144 - 0.322102 | 0.322103 - 0.536130 |
| 2015 | 0.125816 - 0.178079 | 0.178080 - 0.261897 | 0.261898 - 0.555030 |
| 2016 | 0.131963 - 0.170613 | 0.170614 - 0.267632 | 0.267633 - 0.514040 |
| 2017 | 0.128950 - 0.158546 | 0.158547 - 0.277637 | 0.277638 - 0.513120 |
| 2018 | 0.136583 - 0.184832 | 0.184833 - 0.286964 | 0.286965 - 0.558392 |
| 2019 | 0.124588 - 0.206110 | 0.206111 - 0.394530 | 0.394531 - 0.570715 |
| 2020 | 0.131834 - 0.188278 | 0.188279 - 0.337378 | 0.337379 - 0.597113 |
| 2021 | 0.149486 - 0.223305 | 0.223306 - 0.372307 | 0.372308 - 0.604249 |

**Table S2 The ranges of digital economy classification**

| Year | Low-level area | Medium-level area | High-level area |
| --- | --- | --- | --- |
| 2011 | 0.005994 - 0.021506 | 0.021507 - 0.095989 | 0.095990 - 0.195398 |
| 2012 | 0.015007 - 0.033759 | 0.033760 - 0.113603 | 0.113604 - 0.212296 |
| 2013 | 0.024732 - 0.070676 | 0.070677 - 0.185566 | 0.185567 - 0.309638 |
| 2014 | 0.030069 - 0.086871 | 0.086872 - 0.177914 | 0.177915 - 0.317810 |
| 2015 | 0.039394 - 0.093954 | 0.093955 - 0.212936 | 0.212937 - 0.358521 |
| 2016 | 0.047431 - 0.108845 | 0.108846 - 0.253422 | 0.253423 - 0.417251 |
| 2017 | 0.053224 - 0.128999 | 0.129000 - 0.359326 | 0.359327 - 0.515421 |
| 2018 | 0.070679 - 0.126600 | 0.126601 - 0.253680 | 0.253681 - 0.638748 |
| 2019 | 0.083047 - 0.150102 | 0.150103 - 0.293993 | 0.293994 - 0.634392 |
| 2020 | 0.084265 - 0.170117 | 0.170118 - 0.296615 | 0.296615 - 0.668056 |
| 2021 | 0.067694 - 0.173292 | 0.173293 - 0.324748 | 0.324749 - 0.692124 |
